# Comparative Oncology: Management of Hepatic Neoplasia in Humans and Dogs

**DOI:** 10.3390/vetsci9090489

**Published:** 2022-09-08

**Authors:** Erin A. Gibson, Roger E. Goldman, William T. N. Culp

**Affiliations:** 1 Department of Surgical and Radiological Sciences, School of Veterinary Medicine, University of California-Davis, Davis, CA 95616, USA; 2 Department of Radiology, University of California-Davis Medical Center, Sacramento, CA 95817, USA

**Keywords:** liver, hepatocellular, lobectomy, embolization, transarterial

## Abstract

**Simple Summary:**

Hepatocellular carcinoma is identified not uncommonly within the canine and human population. Substantial effort has been put forth to identify treatment strategies that can maximize positive outcomes in people diagnosed with this disease. While the origin of this disease in dogs is different than in people, treatment strategies remain similar, and translatable, across species even if indications for treatment are unique between the two populations. Below is a summary of treatment trends in veterinary medicine regarding canine liver cancer and a comparison to the treatment options utilized in human medicine. Current strategies within each population are compared and explored, which serves as a foundation for future research endeavors. The dog has been considered as a model for novel treatment options in human medicine and the hope is that this will continue to provide benefit to both canine and human populations during the management of hepatocellular carcinoma.

**Abstract:**

Primary hepatic neoplasia is uncommonly reported in dogs. Hepatocellular carcinoma (HCC) is the most frequent neoplasia identified in dogs and considerable effort has been committed towards identifying definitive and palliative treatment options. HCC is well recognized in humans as a sequelae of liver disease such as hepatitis or cirrhosis, while in dogs a similar link has failed to be fully elucidated. Management of HCC in people may be curative or palliative dependent on staging and transplant eligibility. Despite differences in etiology, there is substantial similarity between treatment options for liver neoplasia in human and veterinary medicine. The below summary provides a comparative discussion regarding hepatic neoplasia in dogs and people with a specific focus on HCC. Diagnosis as well as descriptions of the myriad treatment options will be reviewed.

## 1. Introduction

Liver tumors in companion animals are uncommon, accounting for 0.6–1.3% of neoplasms in dogs, with the vast majority occurring in animals over the age of 10 [1]. Primary tumors of the liver, such as hepatocellular adenomas, hepatocellular carcinomas, cholangiocellular carcinomas, and neuroendocrine carcinomas are diagnosed in 15–26% of dogs; however, this occurs at similar or lower rates as metastatic disease in the liver (10–46%) [2,3,4]. Other hepatic tumor types identified, in descending frequency, include bile duct or undifferentiated carcinoma, sarcomas, lymphoma, malignant histiocytosis, and mast cell tumor [2,3].

The most common malignant primary hepatic tumor in dogs is hepatocellular carcinoma (HCC) [1,5]. HCC generally occurs in three forms, including solitary or massive (>50%), multifocal, and diffuse [2,5]. Large, solitary HCC have a very low mitotic rate, which correlates clinically with a low metastatic rate. This is at odds with the nodular and diffuse form, which reportedly have greater mitotic rates and are often metastasized at the time of diagnosis [3]. The most commonly reported location for solitary HCC is the left lateral liver lobe [1,6]. Although the rate of metastasis in clinical cases of HCC is low overall, intra-hepatic extension is the most common form of dissemination followed by extra-hepatic metastasis to the local lymph nodes and lungs [1,5].

Liver cancer in humans is the sixth most common cancer diagnosis and the third most frequent cause of cancer-related death globally [7]. In the United States in 2022, an estimated 41,260 new cases of liver cancer will be diagnosed with a mortality estimate of 30,520 [8]. Globally and within the United States, liver cancer is one of only three cancers to have an increasing mortality rate year-over-year for the past 20 years [8]. Primary liver cancers comprise a heterogeneous group of diseases, with varying histological and prognostic features. In contradistinction to dogs, HCC accounts for approximately 90% of all primary liver cancer in humans [9]. Hepatitis B virus infection is the most prevalent cause of HCC worldwide, accounting for 55% of cases globally and 89% of cases in endemic regions [10]. Other known causes include hepatitis C virus, aflatoxin B1, and alcohol consumption leading to liver cirrhosis. Evidence suggests these disease states lead to an alteration of cell gene expression, likely contributing to carcinogenesis and subsequent disease [10,11]. Similar risk factors in dogs have failed to be identified although hepadnaviruses have been discovered in dogs and cats in association with altered hepatic markers. The significant and causative role in subsequent disease in these species is unknown [12,13]. Identifying similarities and comparing the disease syndromes of HCC in people and dogs will continue to be essential in furthering treatment strategies in humans and animals while acknowledging important differences and possible future directions.

## 2. Diagnostics

Diagnosis of liver tumors can be challenging with cytologic changes existing on a scale of normal to adenomatous to carcinomatous. Imaging, cytology, and histopathology remain key diagnostic entities when evaluating tumors of hepatic origin, while biochemical changes are less essential in understanding this disease entity. Biochemical differences in dogs with benign vs. malignant hepatic tumors have been evaluated. Additionally, serum alanine aminotransferase (ALT) and aspartate aminotransferase (AST) levels have been evaluated for associations with hepatic lesions and clinical outcomes. ALT:AST ratios less than 1 have been associated with HCC and bile duct carcinoma, and ratios more than 1 have been associated with carcinoids and sarcomas. Elevated ALT, AST, and alkaline phosphatase (ALP) have also been associated with a poor prognosis in dogs diagnosed with HCC [14,15]. Serum ferritin is of interest as it has been associated with hepatic injury and may reflect ongoing inflammation and necrosis. Serum ferritin concentration has been found to be significantly higher in dogs with hepatic malignancies compared to those with benign changes. Alpha-fetoprotein (AFP) has been established as an accurate tumor marker for the presence of HCC in people when detected at elevated levels, and may be used as an adjunct for monitoring recurrence [16,17]. This phenomenon has been evaluated in dogs, and similarly high levels of serum AFP (>1400 ng/mL) occurred in the majority of dogs with HCC, while levels that were <70 ng/mL were measured in all clinically healthy dogs [18]. Other tumor types associated with elevated AFP include cholangiocarcinoma and hepatic lymphosarcoma [19]. These findings may support an earlier diagnosis of primary malignant hepatic neoplasms in dogs, although this test has yet to be widely adopted by clinical centers [18,19].

Paraneoplastic syndromes associated with hepatic neoplasms may manifest in various biochemical derangements. The occurrence of hypoglycemia in dogs with hepatic neoplasia is suggested to be secondary to the increased generation of insulin-like growth factor 2 (IGF-2), a normal product of hepatic metabolism that may be secreted in excess with increased tissue (tumor) volume. This has been reported secondary to HCC in dogs, and is also associated with mesenchymal and epithelial tumors in people, which often manifests clinically during the fasting state as neurologic symptomology [20,21]. Hyperalbuminemia, a relatively unrecognized syndrome in dogs, was identified in one dog in conjunction with hepatocellular carcinoma [22], and although similarly rare, has also been documented in people [23]. Additionally, thrombocytosis is recognized as a paraneoplastic syndrome in dogs with liver tumors; interestingly, the most common cause of secondary thrombocytosis in dogs is neoplasia, accounting for 55–56% of cases [24,25] with HCC representing the 4th most common neoplasm [24]. This is comparable to what is found in people, occurring in a significant percentage of patients with HCC, though the etiology of thrombocytopenia reflects sequelae of portal hypertension and splenomegaly, secondary to the cirrhosis with which HCC is predominantly associated [26]. The increased production of thrombopoietin by neoplastic tissue may account for thrombocytopenia in the absence of cirrhosis or in large tumor volumes [26,27]. Severe hypercalcemia secondary to intact parathyroid hormone hypersecretion has been documented in people with HCC. Surgical resection of liver tumors has led to calcium homeostasis normalization in such patients, and similar outcomes have been documented following transarterial embolization, suggesting this as an effective alternative to surgical resection in cases for which paraneoplastic syndromes are documented [28,29,30]. Other paraneoplastic manifestations include hyperthyroxinemia, hypercholesterolemia, hypertriglyceridemia, and erythrocytosis, although these have not been documented in dogs to the authors’ knowledge [23,31]. In humans, a poorer prognosis is associated with onset of paraneoplastic syndromes although, concurrently, onset of such syndromes are associated with greater disease burden and more advanced disease, which are essential contributors to survival [31].

Ultrasonography is regularly utilized as a diagnostic tool for hepatic neoplasms in dogs, and as a screening mechanism in humans [17]. In dogs, solitary masses are associated with a cytologic diagnosis of neoplasia while multifocal nodules are more commonly associated with benign change (vacuolar hepatopathy) [32]. Ultrasound elastography can be used to differentiate tissue density, but this was found to be unhelpful overall in distinguishing between different hepatic neoplasms in a cohort of dogs [33]. Contrast enhanced ultrasound (CEUS) utilizes intravenous contrast to optimally, and in real time, identify tissue vascularity. This may provide additional insight into the tumor type, with patterns of wash-in and wash-out significantly different for well-differentiated HCC, specifically. It is reported to have excellent specificity and sensitivity in identifying hepatic malignancies in dogs [34,35]. In people, ultrasound is a recommended screening modality for patients at high risk of HCC. CEUS protocols and enhancement patterns for various hepatic pathologies are well-described, though they are used sparingly at specialized centers for characterization of indeterminant nodules, have minimal utilization in common practice, and are not recommended for surveillance of staging [17,36,37].

Triple-phase CT and MRI characteristics of hepatic tumors including HCCs, adenomas, and metastatic lesions have been described and form the mainstay of clinical diagnosis in humans [17]. In contradistinction to humans, subtle differences between images of various hepatic pathologies may often be suggestive of a final diagnosis, but accuracy has yet to reach a level sufficient to determine definitive diagnosis in companion animals [38,39]. In dogs with focal liver lesions undergoing delay-phase contrast CT, a decision- making tree has been proposed for differentiating between HCC, nodular hyperplasia (NH), other benign lesions, and other malignant lesions. Five CT features were evaluated, with a reported accuracy of identifying nodular hyperplasia, other benign lesions, HCC, and other malignant lesions of 74%, 88%, 87%, and 75%, respectively. Notably, there was an overall global misclassification rate of 38% [40]. A recent meta-analysis, pooling reports of CT diagnosis of malignant and benign focal liver lesions in dogs, identified several features such as presence of a capsule (defined as a hyperenhancing fibrous peripheral border), hypoattenuation, and heterogeneity in the delayed phase that together led to a high diagnostic odds ratio [41]. While these reports both describe and optimize CT diagnosis of hepatic lesions, cytology and histopathology remain essential in the diagnosis of focal liver lesions in dogs. This is at odds with what is reported in people, where multiphasic CT or MRI retains high enough diagnostic accuracy in many cases such that further, more invasive diagnostics are unnecessary [42]. The recommendations for what is considered diagnostically satisfactory vary according to lesion, but for focal liver lesions suspected to be HCC, CT or MRI demonstrating typical findings of HCC are considered definitive [43,44,45].

Unlike in people, MRI is infrequently utilized for focal liver lesion evaluation in dogs. In one study, MRI was reported to have exceptionally high sensitivity (100%) and specificity (90%) for distinguishing malignant and benign hepatic lesions and was reported to identify HCCs in dogs with accuracy [46]. Recently, focus has turned to the utility of pairing gadoxetic acid, a paramagnetic, hydrophilic, ionic contrast-agent taken up by functioning hepatocytes, with MRI in the diagnosis of focal liver lesions. In dogs, lesions such as nodular hyperplasia, metastatic lesions, adenomas, and carcinomas are reported to share similar imaging characteristics with those in people, for which this modality can provide definitive diagnosis [47,48]. Further investigation into the utility of MRI in focal liver lesion distinction and diagnosis in veterinary medicine is certainly warranted.

## 3. Tumor Sampling

Cytology of liver tumors is unreliable at distinguishing between malignant and benign etiologies in dogs. Cytologic characteristics associated with canine HCC include dissociation of hepatocytes, acinar or palisading arrangements of neoplastic cells, and presence of naked nuclei along with classic features of malignancy (anisocytosis, anisokaryosis, multinuclearity, increased nuclear:cytoplasm ratios) [49]. Advantages of fine needle aspirate (FNA) in the diagnosis of focal liver lesions lie in the minimally invasive nature and low reported mortality (<0.3%) in dogs [3]. Agreement between cytology and histopathologic diagnosis is reportedly low, ranging from 30–60% [50,51,52], and cytology is overall considered to be most accurate in identifying diffuse disease such as vacuolar hepatopathy [50,51]. Importantly, FNA inconsistently identifies hepatic neoplasms, both diffuse (e.g., lymphoma) and focal (e.g., HCC). In one study, only two of 14 (14%) neoplasms were diagnosed correctly using cytology, which included the misdiagnosis of carcinomas, sarcomas, and hematopoietic neoplasms as benign change. Although cytology bears a relatively high positive predictive value for neoplasms, the absence of cytologic change consistent with neoplasia certainly does not rule out the presence of cancer [53]. This highlights the dilemma facing clinicians when selecting diagnostic tools for further evaluation of focal liver lesions, as well as the cautious interpretation that is necessary when evaluating cytologic results of liver aspirates.

Needle biopsy, most often ultrasound-guided, is reported to provide adequate tissue for histopathologic diagnosis in 92–96.3% of cases [54,55]. Importantly, when compared to larger tissue samples, agreement between needle biopsy samples and wedge or necropsy samples ranged from 48–66% [56,57]. In addition to needle biopsy samples, 8 mm punch biopsy and 5 mm cup biopsy samples were compared to a sample obtained during necropsy and similarly low agreement in 69% and 60% of cases, respectively, was encountered [57]. This is an important reminder that although histopathologic diagnoses can be made on needle biopsies (and other sampling methods), achieving accurate and actionable information may be challenging. The major advantage of needle biopsy is the retrieval of larger volumes of tissue compared to a fine needle aspirate while retaining the benefits of minimally invasive acquisition. It should be noted that needle biopsy outperformed wedge biopsy for diagnosis of disorders for which inflammatory change was the most prominent characteristic, but in all other histopathologic findings wedge biopsy outperformed needle biopsy [56]. Acquiring samples with enough portal triads to confer adequate diagnostic utility can be challenging in dogs, which may be a reflection of the anatomic differences between people and dogs, who have fissured and tapering macroscopic characteristics of the liver lobes compared to the brick-like liver of humans. 

The major complication associated with needle biopsy is bleeding from the biopsy site. Ultrasound-guided liver biopsies utilizing 16 gauge and 18 gauge biopsy needles have been retrospectively evaluated, and major hemorrhage (corresponding to a 6% decrease in packed cell volume) occurred in 42% of dogs undergoing needle biopsy. Importantly, dogs with neoplasia were more likely to have a complication, although overall risk of major clinical complications occurred in only 1.9% of dogs [55]. Although the role of needle biopsy in the diagnosis of liver lesions has not been well defined in veterinary medicine, this procedure appears to have some utility and tolerable risk; however, clinicians must weigh the potential risks of such a procedure and likelihood of actionable information.

Laparoscopic liver biopsy is a minimally invasive method of sampling multiple liver lobes and is most commonly performed with 5 mm cup biopsy forceps. The diagnostic accuracy of 3 mm cup biopsy forceps has been evaluated in canine cadavers and found to be comparable to 5 mm cup biopsy forceps [58]. Pre-tied ligature loops have also been evaluated and found to acquire more tissue volume with less surgical artifact, although procedural duration was longer than with standard cup biopsy forceps [59]. In veterinary medicine, laparoscopic liver biopsy is most commonly utilized for lesions located peripherally that may be more representative of nodular or diffuse disease, although it does have utility in neoplasia diagnosis [60,61]. The major complication (such as bleeding requiring transfusion or conversion to open approach) rate is reported in 1.9–7.5% of dogs [60,61]. Ultimately, liver biopsy with a variety of methods (ligature, biopsy punch, biopsy needle, laparoscopic, and ultrasonically activated scalpel) is considered safe [62].

## 4. Surgical Treatment of Hepatic Neoplasia

Surgical treatment of primary liver tumors is pursued most commonly when masses are focal, non-metastasized, and occupying a resectable region of the liver. Importantly, experimental studies have established that normal dogs can tolerate up to 70% single session hepatectomy while retaining liver function and appropriate portal perfusion [63,64]. Beginning as soon as 1 day post-operatively, substantial compensatory hypertrophy and hyperplasia of hepatocytes occurs, leading to an average regeneration of 113% of pre-lobectomy liver volume in dogs [63]. In people, focus has turned to evaluating the volume of the future liver remnant (FLR) rather than the volume of tissue excised. Importantly, smaller FLR is associated with longer hospital stays and liver function derangements [65,66,67]. Pre-surgical portal vein embolization (PVE) may be utilized to promote selective hypertrophy in patients in which an unaugmented FLR portends a poor prognosis, such as an FLR < 20% in a normal liver and <40% in a cirrhotic liver [65,66,67]. PVE is the selective attenuation of portal blood supply to the liver segments that will be resected in the future, leading to compensatory hypertrophy of the non-embolized liver prior to surgery. By allowing for pre-operative hypertrophic compensation, the functional liver remnant may be better able to adjust to changes in blood flow that necessarily follow large liver resections, and preserve adequate function [66]. This has shown to be effective in significantly increasing FLR prior to major hepatectomy and can also lead to shrinkage of the planned resected tissue [65,66,67]. Embolization of the left portal vein has been done experimentally in normal dogs with coil-gelfoam embolic, as well as absolute ethanol embolic. In that study, there were no reports of hepatic dysfunction or portal hypertension in the treatment groups and liver volume of the FLR increased up to 33.2% compared to pre-embolization values [68]. PVE has yet to be repsorted in clinical canine cases, although experimental success suggests that PVE may be considered if the appropriate clinical indication arises. 

As HCCs occur most commonly in the massive form and remain the most common primary tumor affecting the canine liver, HCCs are often treated surgically. This is at odds with what is reported for people, for which the occurrence of this tumor is associated with, most commonly, chronic alcohol consumption, hepatitis B, hepatitis C and non-alcoholic fatty liver disease [7,8,9,10,11]. As a result of these tumors occurring in a state of global hepatic disease and dysfunction, it can be difficult to identify early and, as such, curative-intent liver resections apply to a small (20–30%) subset of humans diagnosed with this disease [42,45,69,70]. In dogs, certain patient, owner, and disease factors may prohibit surgical treatment or resection of massive HCCs. Non-treatment compared to surgical treatment, however, has been shown to lead to vastly different outcomes, with surgically treated dogs achieving median survival times of >1460 days compared to non-treated dogs achieving median survival times of 270 days. Importantly, the tumor-related mortality rate was 15.4 times higher in dogs in the non-surgery group [14]. With this in mind, seeking definitive surgical care for dogs with massive HCCs is compelling. 

Overall, the nodular and diffuse forms of HCC are considered to have a poorer prognosis [1,71]. Traditional metronomic chemotherapy has in rare instances been associated with prolonged survival [72], although generally has yet to prove substantial effectiveness as a treatment for gross HCC [73]. In humans with inoperable disease, who are not candidates for locoregional therapy, systemic therapies, including the preferred regimens of immunotherapy, are recommended by multiple societies [74,75,76]. Treatment with sorafenib compared to metronomic chemotherapy for unresectable HCC has also been reported in dogs. Patients in the sorafenib group demonstrated median time to tumor progression of 363 days compared to 27 days in the metronomic group; however, larger clinical cohorts will be essential for evaluating this promising treatment [71]. Survival benefits have been demonstrated in people with HCC treated with sorafenib as well, motivating further research in sorafenib in combination with metronomic chemotherapy for improving tumor control [77]. 

**Figure 1:**
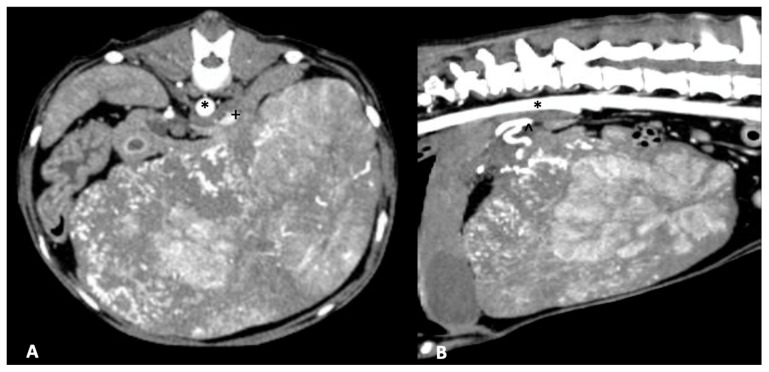
Massive hepatocellular carcinoma arising from the right lateral liver lobe in an 8-year-old mixed-breed dog. The patient underwent right lateral liver lobectomy without complication. (**A**) Arterial phase CT image in axial plane of the right lateral liver hepatocellular carcinoma with the aorta (asterisk) and compressed caudal vena cava (plus sign) demarcated. (**B**) Arterial phase CT image in sagittal plane with the aorta (asterisk) and hepatic artery (caret) demarcated.

**Figure 2:**
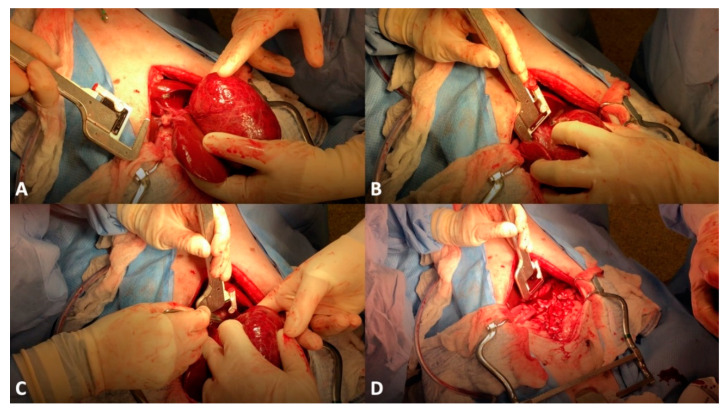
Left lateral liver lobectomy in a 11-year-old mixed-breed dog. (**A**) The liver mass (hepatocellular carcinoma) has been exteriorized to allow for isolation of a pedicle. (**B**) A thoracoabdominal stapler has been placed at the level of the liver lobe hilus. (**C**) After compression of the stapler cartridge, and release of the staples, a scalpel blade is being used to transect the liver lobe at the level of the hilus. (**D**) The liver tumor has been removed.

Surgical techniques for liver lobe tumors described in small animals include partial liver lobectomy, or total/hilar liver lobectomy. In dogs, there is a consistent single hepatic artery and biliary duct associated with each liver lobe although there may be multiples of portal and hepatic veins, highlighting the importance of a pre-operative CT scan (Figure 1) for appropriate planning [78]. Additionally, the length and location of the central hepatic vein in dogs is frequently prohibitive of an independent resection of the right medial or quadrate liver lobes, necessitating en bloc removal of the central division if either lobe is affected [78]. Surgical stapling and blunt dissection and ligation have been compared for hilar liver lobectomy, with both achieving similar outcomes regarding risk of hemorrhage, although the ligation technique had significantly increased surgical times compared to stapling [79]. Methods of partial liver lobectomy include stapling, blunt dissection and ligation, skeletonization and vascular clipping, use of energy devices (e.g., vessel-sealing device, ultrasonic devices), and pre-tied endoscopic loops. Increased risk of hemorrhage has been associated with skeletonization and vascular clipping when compared to the other techniques [80]. Leakage of surgical sites perfused with supraphysiologic pressures in dogs following partial liver lobectomy identified pre-tied ligature as the most secure vascular ligation, although none of the techniques listed above demonstrated leakage at physiologic pressures [80]. Clinically, a combination of techniques may be necessary to navigate the unique vascular and parenchymal anatomy encountered during hepatobiliary surgery for each patient (Figure 2). 

Surgical treatment of HCC in dogs bears an overall good prognosis, with perioperative survival reported in 92–93% [6,14,52] of cases and excellent long-term outcomes achieved by the majority of patients [6,14]. Complications of surgical resection of liver tumors include major hemorrhage [6,14,81], with reports of intraoperative exsanguination occurring from vascular (often caudal vena cava) trauma [14,82]. Inadvertent occlusion of the caudal vena cava has also been reported [6]. While left liver lobectomy is the most common procedure performed for curative-intent surgery, central division lobectomies or right sided liver lobectomies are associated with a higher risk of intra-operative complications such as major hemorrhage [52,82]. Within the cohort of central division liver lobectomies, substantial hemorrhage is reported in up to 33% of dogs and of those, 95% required transfusions [82]. This is in contrast to surgical excision of other lobes, for which the risk of transfusion is less (9%) [52]. Reported perioperative mortality rates for central-divisional or right-sided liver lobectomies range from 11–40% although 1- and 3-year survival rates may be reached by the majority of survivors (82.1%) [82]. This highlights the need for different levels of surgical preparedness depending on the location of the tumor, although the pursuit of surgery as a curative-intent option for liver tumors remains ideal in companion animals. Laparoscopic liver lobectomy has been reported in cadaver dogs and six client-owned dogs with varying success and was found to be ideally suited for small, peripheral tumors of the central and left liver divisions [83]. 

Tumor size has been weakly associated with poor prognosis in dogs, with tumor size of >5 cm identified as an adverse prognostic factor for hepatobiliary tumors [15,84]. This finding is not corroborated by all studies, and as such, should not prohibit or alter treatment options on the basis of perceived prognosis [15]. In dogs surgically treated for left-sided HCCs, there were significantly better survival times compared to dogs with right-sided HCC, although this may be more reflective of the surgical challenge inherent to the right side of the liver [14]. Incomplete resection has been associated with shorter progression-free survival and overall survival compared to complete resection of HCC in dogs, although survival times of over two years may be achieved in both groups [85]. Other studies have not identified completeness of resection with a difference in prognosis or outcome in dogs [14,52,82], and concurrently report overall survival times of >2 years. It should be noted that evaluating the surgical margin for completeness of HCC resection is inherently challenging, and close monitoring for tumor recurrence is warranted if excision is incomplete. Recurrence rates range from 0–58% [6,14,85], although the rate of tumor-related death is reportedly low, ranging from 0–40% [6,14,85].

Various staging systems have been evaluated for accurate prognostic assessment and may be used to guide treatment decision-making including surgical, locoregional, and systemic therapy options [17]. A Child–Pugh (CP) score is assigned in humans with liver tumors and is based on a combination of liver function tests in addition to the presence of ascites and encephalopathy. Lower CP scores define better liver function and lower operative risk, and hepatic resection is typically reserved for patients retaining liver function defined by CP Class A [86,87], noting highly selected patients with Child–Pugh Class B liver function may be eligible for limited resection [17]. 

Indocyanine green (ICG) is a dye that is actively taken up by hepatocytes and secreted unchanged into bile [87,88]. ICG retention tests can be used to predict parenchymal function and hepatic blood flow, and have been found to accurately predict portal hypertension and presence of esophageal varices in cirrhotic patients although this diagnostic is performed uncommonly [88]. These tests may be used in conjunction with CP scores to predict liver function and subsequent appropriateness of hepatic resection. Additionally, the Milan criteria, an imaging subset of the United Network for Organ Sharing (UNOS) criteria, has been used to identify triage patients for which orthotopic liver transplantation (OLT) is appropriate. A single HCC less than 5 cm, or three HCC lesions less than 3 cm without angioinvasion or extrahepatic involvement satisfy this criteria and allow patients to be considered for transplantation. 

Orthotopic liver transplantation is the substitution of a diseased native liver with a normal donor liver. When performed in people meeting UNOS criteria, this procedure has led to excellent short- and long-term survival with uncommon (<10%) tumor recurrence. Due to the infrequency of early detection of HCC satisfying the Milan criteria, downstaging of advanced HCC with locoregional therapies such as ablation, arterially-directed therapies, or external beam radiation therapy, have been explored with mixed results [89]. Similar “bridging” therapies have been evaluated for patients who satisfy Milan criteria required for OLT but who may suffer disease progression prior to receiving definitive care. The exact role for these therapies continues to be investigated [89].

Surgical treatment of liver tumors in people is most commonly performed for secondary metastasis, followed by primary hepatic malignancy, biliary tract malignancy, and benign hepatic tumors [90]. Mortality rate for hepatic resection is reported to be near 5% [90]. Specific to HCC, taking “wide” margins is recommended. In early stage HCC, surgical margins of >2 mm were found to be associated with a decreased risk of recurrence and improved overall survival [51]. This is compared to treatment of HCC with partial hepatectomy for which surgical margins of 2 cm were found to decrease recurrence and improve survival compared to 1 cm margins [90]. While achieving wide margins is considered optimal, the definition of “wide” surgical margins has yet to be universally determined. Importantly, the long-term outcome following surgical resection in patients with advanced liver cirrhosis is poor due to the recurrent carcinogenesis of remnant liver in people, justifying the rigorous pre-surgical criteria that has been established [91].

Laparoscopic hepatic resections are performed in people, with similar survival and disease-free survival rates to those reported with open procedures. Additionally, minimally invasive hepatectomy is associated with shorter hospitalization stays as compared to open hepatectomy [87]. Indications for laparoscopic hepatectomy include small, well-demarcated nodules peripherally located, with specific ease reported in the lateral segment and segments 4–6. Furthermore, robotic hepatectomy has been reported to be safe and effective compared to open surgery [92]. Both laparoscopic and robotic hepatectomy are considered relatively new interventions and further randomized controlled trials are required to formalize the indications for these therapies in people [89,92].

## 5. Interventional Management of Hepatic Neoplasia

Alternatives to traditional surgical excision of liver tumors such as percutaneous thermal ablation, arterially-directed therapies, and external beam radiation are regularly considered in humans ineligible for surgical therapy, depending on various staging schemes [70]. Percutaneous ablation has performed comparably to surgical resection for disease-free survival, recurrence-free survival, and overall survival in people with small lesions and early-stage disease. Transarterial bland embolization (TABE) or chemoembolization (TACE) is typically reserved for palliative-intent treatment or as a bridge to transplantation [70,93]. Chemoembolization is the delivery of intra-arterial chemotherapy in conjunction with embolization to elevate intratumoral drug levels. Conventional TACE generally involves the combination of embolic, lipiodol, and chemotherapeutic. Lipiodol has the unique feature of being drug-carrying and tumor-seeking, and is absorbed and confined to the hepatic neoplastic tissue for months to a year [93]. Transarterial radioembolization (TARE) with Yttrium-90 (Y^90^) has also been evaluated in the context of curative-intent or palliation. For small HCC lesions, Y^90^ TARE has demonstrated comparable outcomes to ablation, resection, and transplantation [70,94]. External beam radiotherapy has also been performed in people to treat HCC. Concerns regarding efficacy and safety have limited the use of this modality historically, although newer three-dimensional conformal intensity-modulated and stereotactic body radiotherapy have allowed for more focused treatment plans. Early reports suggest efficacy for improving local progression and this may be considered as an alternative to ablation techniques although further evidence is required in order to assign a consistent clinical role to this modality [70,95]. Intensity-modulated stereotactic body radiotherapy (IM-SBRT) has been reported in one dog for treatment of an incompletely resected HCC, for which tumor regression was reported in the 10 months of follow-up available [96]. Three-dimensional conformal radiation therapy, considered less tissue-sparing than IM-SBRT plans, has been reported for treatment of non-resectable massive HCC in six dogs. This was reported to achieve partial response in five dogs, with radiation-induced side-effects identified in only one patient, and an overall median survival time of 567 days [97]. Similar to the role in people, radiation therapy for non-resectable liver tumors in dogs is incompletely defined and, at this stage, reserved as a palliative therapy if other curative-intent modalities such as surgical resection are not feasible.

**Figure 3:**
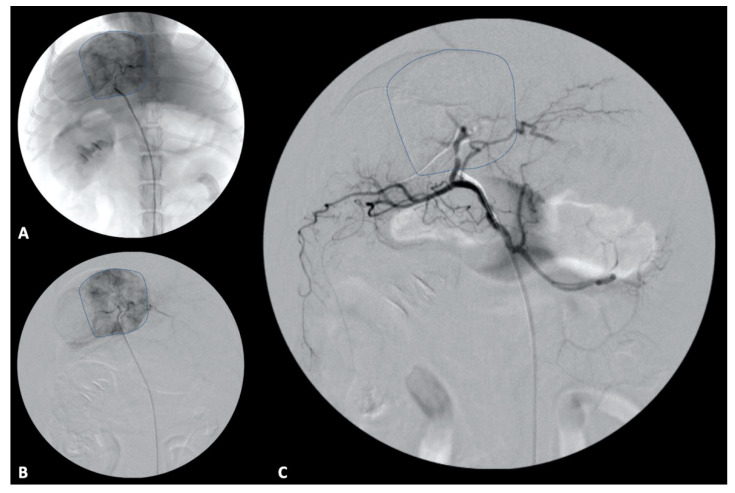
Transarterial embolization in a 13-year-old mixed breed dog. (**A**,**B**) An angiographic catheter has been placed in the hepatic artery and a direct angiogram of the hepatic tumoral blood supply has been performed. The liver tumor is delineated by a hashed line on both non-subtracted (**A**) and subtracted (**B**) angiographic images. (**C**) After embolization, the liver tumor is non-enhanced as the tumoral blood supply has been eliminated.

TAE and TACE have been evaluated in the treatment of canine hepatobiliary and prostatic tumors most commonly [98,99]. Embolization is the intravascular delivery of a device or materials, such as polyvinyl alcohol (PVA) or hydrogel polymers, to interrupt blood flow. Prior studies have validated the concept of intra-arterial drug infusions leading to higher concentrations in downstream tissues compared to intravenous infusions while sparing non-target tissues [100]. Drug eluting beads (DEB) are a novel method of TACE, providing a delivery system (beads) of chemotherapy in a sustained release form [101,102]. Potential benefits of DEB-TACE include lower systemic levels of chemotherapy (doxorubicin) in patients undergoing DEB-TACE compared to conventional TACE, which may enhance patient safety [103]. Evaluation of DEB with doxorubicin identified systemic doxorubicin levels that were unmeasurable 30 min after delivery within the tumor bed to stasis [104], and there was no systemic chemotherapy detected following deployment of DEB loaded with cisplatin into the left hepatic artery of normal dogs [105]. These findings demonstrate the substantial potential benefit in sparing systemic exposure of dogs to chemotherapy when treated with DEB-TACE. Clinical performance of DEB-TACE with doxorubicin in dogs with non-resectable HCC achieved stable disease (62%) or partial response (23%) at a median of 74 days following treatment. Minor and major complications occurred at a frequency of 26 and 11% respectively, and median survival time was 337 days [106]. Transarterial embolization and TACE for treatment of non-resectable hepatic tumors in dogs have been uncommonly reported [99,107,108,109] (Figure 3). The outcomes of two dogs with non-resectable HCC treated with TACE identified stable disease at 1 month, and progressive disease at 3 months, although superselection of tumor arterial supply was not achieved in either case [108]. In a separate report, TACE performed in four dogs with paclitaxel-containing embolic microspheres lead to a median tumor volume decrease of 42% at 1 month post treatment [109]. 

Complications of TAE, TACE, and DEB-TACE in people include hemorrhage at the vascular site, non-target embolization, hepatic abscess formation/infarction, renal failure, cholecystitis/rupture, and post-embolization syndrome. Post-embolization syndrome, characterized by abdominal pain, fever, or nausea may occur in 36–81% of patients and is associated more commonly with large or multiple tumors, and administration in proximal aspects of the hepatic vascular tree [101,102,110]. Major complications are reported following TAE or TACE in people at a rate of 2.1–4.2%, and the mortality rate for patients suffering major complications is 16.7% [110,111]. Reports in veterinary medicine are less frequent, but the rate of major complication for DEB-TACE of dogs with unresectable HCC was 11%, with overall treatment-associated mortality rate of 7% of dogs [106]. Post-embolization syndrome has been evaluated for prognostic significance and is associated with an increased risk of death in people [110]. A similar association has not been established in veterinary patients, although the presence of vague, flu-like symptoms consistent with a post-embolization syndrome has been appreciated at a frequency of 26–100% of patients [106,107,108,109]. Ultimately, the role of TAE, TACE, and DEB-TACE in dogs with HCC is still being determined, although preliminary reports of its performance in the face of non-resectable HCC is encouraging. Additional clinical reports of safety and efficacy are warranted in order to achieve consensus on the application of this treatment modality in dogs.

Ablation techniques, both chemical and thermal, have been utilized in the treatment of hepatic neoplasia. Percutaneous ethanol injection is one of the earliest devised methods of chemical tumor ablation, although certain weaknesses of this procedure have led to utilization of thermal ablation techniques when clinically available [112]. Microwave ablation uses high-frequency electromagnetic energy to agitate water molecules, leading to frictional heat and coagulative necrosis of the tissues in which it is applied. Benefits of MWA when compared to other ablation techniques such as RFA include faster heating, less susceptibility to heat-sink effect and effective propagation through tissues with high impedance [112,113]. Microwave ablation has been applied to lesions ranging from 0.5–2.5 cm in size in dogs with diffuse hepatic neoplasia; no procedural complications were reported. Due to the variety of diseases treated (biliary adenocarcinoma, hemangiosarcoma, hepatocellular carcinoma, and metastatic apocrine gland adenocarcinoma), long- term outcomes, survival, or disease progression benefits could not be elucidated [114]. Microwave ablation has also been applied to extra-hepatic canine tumors/lesions with similar results reported for lesions ranging from 1.4–3.3 cm in size [98,115]. Radiofrequency ablation uses alternating current to induce frictional heat at the tip of the electrode, leading to coagulative necrosis and cell death [112]. In people, complete ablation is reported to exceed 90% of targeted lesions after two treatments, and local progression after complete ablation is rare [112]. In companion animals, percutaneous ultrasound-guided RFA for primary hyperparathyroidism has been described, leading to resolution of hypercalcemia in all dogs with no major complications reported; however, reports of liver RFA are lacking [116]. Experimental studies in dogs have been performed for evaluating safety and efficacy of RFA. Such studies have identified minimum safe distance from RFA electrode and intrahepatic bile ducts (5 mm) in the treatment of liver tissue [117]. Additionally, canine models have demonstrated tissue coagulation diameter variance with varied impedance and blood flow occurring in different tissue types [118]. To the knowledge of the authors, RFA has yet to be reported in clinical cases for treatment of canine HCC and further research within this area is warranted, although translation from experimental studies may guide future use of this modality. Cryoablation and high-intensity focused US ablation are other ablation options that are much less commonly reported for the treatment of HCC in people, and both modalities have yet to attain a consistent clinical role in the management of this disease [113]. 

## 6. Conclusions

The diagnosis and treatment of hepatic neoplasia in dogs is a fairly well-described entity, although there has been significant evolution over the last 20 years. Achieving good long-term outcomes in dogs is dependent on various patient factors as well as access to and comfort with various diagnostic and procedural modalities. Although HCC is relatively common in both people and dogs, the biologic origin of these tumor types appears to be different, and continues to be investigated in dogs and humans. An interesting overlap in etiology has been recently proposed and may provide additional validity in comparing and contrasting these disease syndromes between species. Advances in human medicine regarding treatment of hepatobiliary neoplasia have been founded on various experimental canine studies and translation of treatment modalities and decision-making schemes between human and veterinary medicine may be cautiously approached moving forward. 

## Data Availability

Not applicable.
